# Identification of Novel Thiazolo[5,4-*b*]Pyridine Derivatives as Potent Phosphoinositide 3-Kinase Inhibitors

**DOI:** 10.3390/molecules25204630

**Published:** 2020-10-12

**Authors:** Liang Xia, Yan Zhang, Jingbo Zhang, Songwen Lin, Kehui Zhang, Hua Tian, Yi Dong, Heng Xu

**Affiliations:** 1 State Key Laboratory of Bioactive Substance and Function of Natural Medicines, Institute of Materia Medica, Chinese Academy of Medical Sciences and Peking Union Medical College, Beijing 100050, China; xialiang@imm.ac.cn (L.X.); zhangyan@imm.ac.cn (Y.Z.); zhangjingbo@imm.ac.cn (J.Z.); linsongwen@imm.ac.cn (S.L.); kehuizhang@imm.ac.cn (K.Z.); tianh@imm.ac.cn (H.T.); 2 Beijing Key Laboratory of Active Substances Discovery and Druggability Evaluation, Institute of Materia Medica, Chinese Academy of Medical Sciences and Peking Union Medical College, Beijing 100050, China

**Keywords:** heterocycle, thiazolo[5,4-*b*]pyridine, phosphoinositide 3-kinase, inhibitory potency, docking analysis

## Abstract

A series of novel 2-pyridyl, 4-morpholinyl substituted thiazolo[5,4-*b*]pyridine analogues have been designed and synthesized in this paper. These thiazolo[5,4-*b*]pyridines were efficiently prepared in seven steps from commercially available substances in moderate to good yields. All of these *N*-heterocyclic compounds were characterized by nuclear magnetic resonance (NMR) and high-resolution mass spectrometry (HRMS) analysis and tested for phosphoinositide 3-kinase (PI3K) enzymatic assay. The results indicated that these *N*-heterocyclic compounds showed potent PI3K inhibitory activity, and the IC_50_ of a representative compound (**19a**) could reach to 3.6 nm. The structure−activity relationships (SAR) study showed that sulfonamide functionality was important for PI3Kα inhibitory activity, and 2-chloro-4-florophenyl sulfonamide (**19b**), or 5-chlorothiophene-2-sulfonamide (**19c**) showed potent inhibitory activity with a nanomolar IC_50_ value. The pyridyl attached to thiazolo[5,4-*b*]pyridine was another key structural unit for PI3Kα inhibitory potency, and replacement by phenyl lead to a significant decrease in activity. Enzymatic Inhibition results showed that compound **19a** inhibited PI3Kα, PI3Kγ, or PI3Kδ with a nanomolar IC_50_ value, but its inhibitory activity on PI3Kβ was approximately 10-fold reduced. Further docking analysis revealed that the *N*-heterocyclic core of compound **19a** was directly involved in the binding to the kinase through the key hydrogen bonds interaction.

## 1. Introduction

Heterocycles are important organic chemical structures commonly found in a large number of agrochemicals, materials, and pharmaceutical molecules [1,2,3]. Heterocyclic structural units are especially prevalent in synthetic drug molecules because they can improve the interaction between drug molecules and proteins and regulate the physicochemical properties of drugs [4,5,6]. It is estimated that about 70% of pharmaceutical products contain heterocyclic substructures, which clearly shows that heterocycles play an extremely important role in drug development and discovery [7,8].

**Figure 1:**
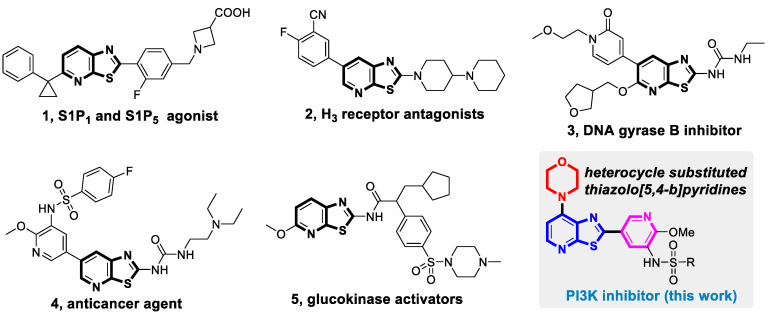
Selected biological compounds with thiazolo[5,4-*b*]pyridine skeleton.

Over the past few decades, many biological compounds containing bicyclic heterocycles have been found with potent pharmacological activities; moreover, some of them have also been successfully developed into drugs approved for marketing [9,10]. As a typical [5,6]-fused bicyclic scaffold, thiazolo[5,4-*b*]pyridine has been regarded as an important privileged structure in medicinal chemistry because of its structural similarity to thiazolo[4,5-*d*]pyrimidine, which is a classic biologically useful skeleton [11]. Furthermore, thiazolo[5,4-*b*]pyridine analogues have been found to exhibit a range of biological activities, such as S1p1 and S1p5 agonist (**1**) [12], H_3_ receptor antagonist (**2**) [13], DNA gyrase B inhibitor (**3**) [14], anticancer agent (**4**) [15], and glucokinase activator (**5**) [16], among others (Figure 1). A few of them show promising drug-like properties, which has led to increased interest in the design, synthesis, and bioactivity evaluation of thiazolo[5,4-*b*]pyridine analogues. In this paper, the novel 2-pyridyl, 4-morpholinyl substituted thiazolo[5,4-*b*]pyridine has been designed and utilized as a template to afford multi-heterocyclic phosphoinositide 3-kinase (PI3K) inhibitors with high potency.

**Figure 2:**
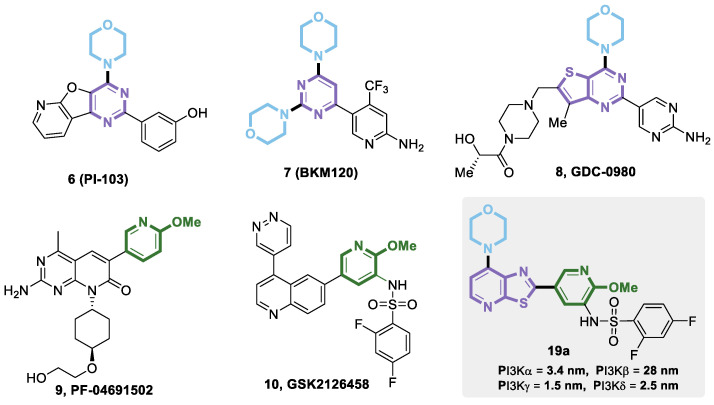
Design of 2-pyridyl, 4-morpholinyl substituted thiazolo[5,4-*b*]pyridine PI3K inhibitor.

The PI3Ks signaling pathway plays a crucial biological function in the process of cell growth, survival, proliferation, and differentiation, which has been proven to be an important target for tumor-targeted therapy [17]. In many tumor cells, the amplification, overexpression, or activating mutation of PI3Kα can lead to abnormal activation of the PI3K signaling pathway, which is a key driving force for tumor occurrence. Given that the PI3K signaling pathway is closely related to tumorigenesis, chemotherapy by inhibiting the PI3K signaling pathway has become an important means of tumor treatment [18], and some potent PI3K inhibitors have been discovered in recent years. It is worth noting that heteroaryl morpholines are common structural cores of PI3K inhibitor molecules—such as PI-103 (**6**) [19], BKM120 (**7**) [20], and GDC-0980 (**8**) [21]—and are representative PI3K inhibitors for the treatment for cancer. Another important core is the 2-methoxyl pyridine unit, which has been seen in PF-04691502 (**9**) [22] and GSK2126458 (**10**) [23] as PI3K/mTOR dual inhibitors. In this paper, we designed a new structural template consisting of morpholinyl and 2-methoxyl pyridine to obtain a series of thiazolo[5,4-*b*]pyridines with high PI3K inhibitory activity. Further analysis of the isoform inhibitory selectivity showed that compound **19a** showed highly potent inhibition of PI3kα, PI3kγ, and PI3kδ with an IC_50_ of 3.4 nM, 1.8 nM, and 2.5 nM, respectively. Furthermore, the IC_50_ value of PI3kβ was approximately 10-fold higher than the other three (Figure 2).

## 2. Results

### 2.1. Synthesis

**Figure 3:**
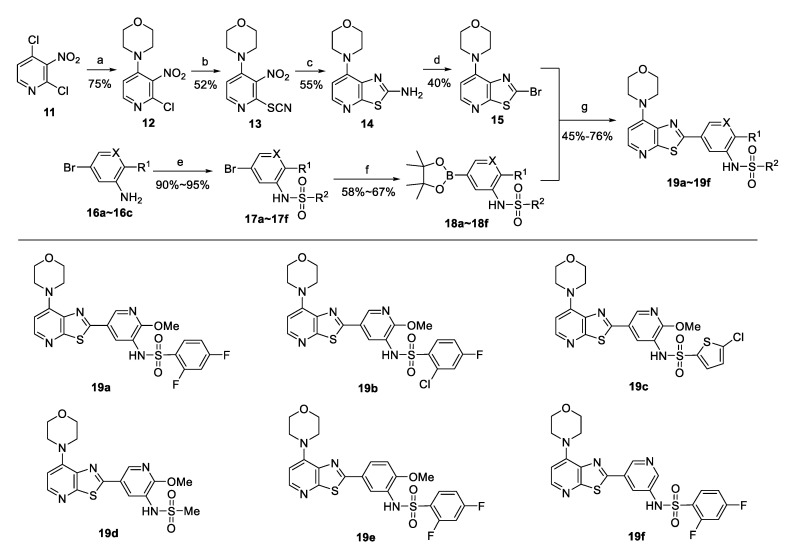
Synthesis of thiazolo[5,4-*b*]pyridine analogues. (**a**) Morpholine, TEA, THF, 0 °C. (**b**) KSCN, HOAc, 80 °C. (**c**) Fe powder, HOAc, 60 °C. (**d**) CuBr_2_, tert-butyl nitrite, CH_3_CN, rt. (**e**) DMAP, pyridine, DCM, rt. (**f**) PdCl_2_(dppf), KOAc, bis(pinacolato)diboron, 1,4-dioxane, 100 °C. (**g**) PdCl_2_(dppf), K_2_CO_3_, 1,4-dioxane/H_2_O, aryl borate, 100 °C. dppf = 1,1′-Bis(diphenylphosphino)ferrocene.

An effective and economical method for preparing the thiazolo[5,4-*b*]pyridine analogues is shown in Figure 3. A commercially available and inexpensive 2,4-dichloro-3-nitropyridine (**11**) was used as a starting material, and the designed seven-step synthetic route was simple and easy to handle. 2,4-dichloro-3-nitropyridine (**11**) could be smoothly transformed into 4-morpholinyl pyridine derivative **12** via selective substation with morpholine in the presence of triethylamine. Molecule **12** underwent another substitution with a thiocyanate by treatment with KSCN in acetic acid at 80 °C to successfully afford 4-(3-nitro-2-thiocyanatopyridin-4-yl)morpholine (**13**). The nitro group was reduced by treatment with Fe powder in acetic acid at 60 °C; the subsequent intramolecular cyclization occurred in one-pot to construct the thiazolo[5,4-*b*]pyridine skeleton, and the corresponding amino thiazolo[5,4-*b*]pyridine derivative (**14**) was obtained in moderate yield. A copper bromide mediated bromination effectively gave the bromothiazolo[5,4-*b*]pyridine derivative (**15**) at room temperature, which underwent a Suzuki reaction with aryl borates at 100 °C to afford the desired heterocycle substituted thiazolo[5,4-*b*]pyridine analogues (**19a**–**19f**) in good yields. Sulfonamide substituted aryl borates were prepared from the corresponding amino-substituted aryl bromide through sulfonamidation and boronization in two steps.

### 2.2. Enzymatic Assay

All of the *N*-heterocycle substituted thiazolo[5,4-*b*]pyridine analogues were evaluated for their PI3Kα inhibitory activity through enzymatic assay. The structure−activity relationships (SAR) are summarized in Table 1. We firstly designed and synthesized an *N*-heterocyclic compound (**19a**) composed of methoxypyridine and morpholinyl thiazolo[5,4-*b*]pyridine. As expected, this thiazolo[5,4-*b*]pyridine compound exhibited extremely strong PI3kα inhibitory activity with an IC_50_ of 3.6 nM. In further SAR studies, the sulfonamide functionality was proved to be a key structural unit that affected activity. For example, despite a 2–3 fold drop in potency compared with that of 2,4-difluorophenyl sulfonamide, 2-chloro-4-florophenyl sulfonamide (**19b**), or 5-chlorothiophene-2-sulfonamide (**19c**) showed high inhibitory activity with an IC_50_ of 4.6 nM and 8.0 nM, respectively. This activity was attributed to the fact that the electron-deficient aryl group resulted in a more acidic sulfonamide NH proton being able to make a stronger charged interaction with Lys802 in PI3Kα [24]. Consistent with this finding, when 2,4-difluorophenyl was replaced with methyl (**19d**), the PI3Kα inhibitory potency dropped over 10-fold (IC_50_ = 53 nM). Removal of the methoxyl group attached to the pyridine ring lead did not significantly affect the potency (IC_50_ = 4.0 nM). Replacement of pyridyl with phenyl resulted in an apparent decrease in PI3Kα inhibitory activity (IC_50_ = 501 nM), demonstrating that the pyridyl attached to thiazolo[5,4-*b*]pyridine was a necessary moiety for enzymatic potency. We also evaluated the inhibitory selectivity of compound **19a** against four isoforms of class I PI3K (Table 2). It potently inhibited PI3Kα, PI3Kγ, and PI3Kδ with nanomolar IC_50_ values, which is approximately 10-fold higher than that of PI3Kβ, clearly proving that it is a novel and potent PI3K inhibitor.

### 2.3. Molecular Docking Study

**Figure 4:**
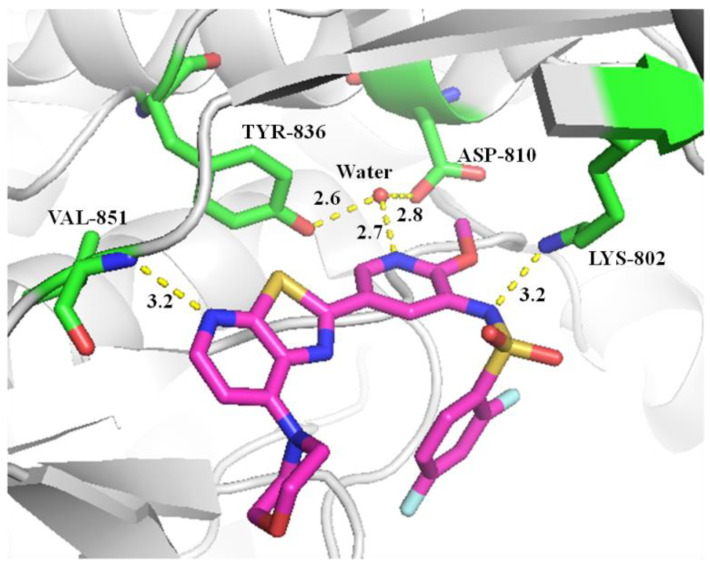
Predicted binding mode of **19a** with PI3Kα.

Because of its superior PI3K potency, thiazolo[5,4-*b*]pyridine **19a** was selected for a molecular docking study. As shown in Figure 4, the 2-pyridyl thiazolo[5,4-*b*]pyridine scaffold fits well into the ATP binding pocket of the PI3Kα kinase, forming a hydrogen bond interaction with the Val851 residue of the hinge region and a water bridge with Typ836 and Asp810. It is worth noting that another hydrogen bond interaction was observed between the sulfonamide group and Lys802. These interaction networks are the key factors for the PI3K bonding of these pyridyl thiazolo[5,4-*b*]pyridine analogues.

## 3. Materials and Methods

### 3.1. General

Acetic acid, 1,4-dioxane, acetonitrile and dried THF were purchased from domestic corporations and used without further purification. Aryl borates were synthesized according to our previous work [24]. Nuclear magnetic resonance spectroscopy (^1^H NMR and ^13^C NMR) was performed on Bruker Advance 400M NMR spectrometers, and high-resolution LC-MS was carried out by Agilent LC/MSD TOF. Purity of all compounds tested for PI3K activity were determined to be >95% by LCMS analysis. The kinase inhibitory activity assay was performed by Shanghai ChemPartner Co., Ltd. (Shanghai, China). For details, see our previously published protocols [24]. Molecular docking study was conducted in the Schrodinger software.

### 3.2. Synthesis

#### 3.2.1. Synthesis of 4-(2-chloro-3-nitropyridin-4-yl)morpholine (**12**)

Morpholine (50 mmol, 4.36 mL) was added dropwise to the mixture of 2,4-dichloro-3-nitropyridine (**11**, 50 mmol, 9.6 g) and trimethylamine (50 mmol, 7.0 mL) in THF (100 mL) at 0 °C, then the reaction mixture was stirred at 0 °C for 1 h, and the reaction was monitored by LC-MS. When the starting material was consumed completely, solvent was removed under reduced pressure, and the residue was purified with flash column chromatography on silica gel to afford compound **7** (9.1 g, 75% yield). Yellow solid, R_f_: 0.5 (EtOAc/Petroleum ether = 1:1), m.p.: 138–140 °C. ^1^H NMR (400 MHz, DMSO-*d6*) δ 8.22 (d, *J* = 6.0 Hz, 1H), 7.25 (d, *J* = 6.1 Hz, 1H), 3.69–3.64 (m, 4H), 3.25–3.20 (m, 4H). ^13^C NMR (101 MHz, DMSO-*d6*) δ 149.9, 149.7, 142.3, 128.0, 113.9, 65.5, 48.6. HRMS (ESI): *m/z* (M + H^+^) calcd for C_9_H_11_ClN_3_O_3_, 244.0483, found: 244.0492. IR (ATR): 3155, 2965, 2865, 1524, 1356, 1215, 1117, 964, 848, 719 cm^−1^. The data is consistent with the data reported in the literature. [25]

#### 3.2.2. Synthesis of 4-(3-nitro-2-thiocyanatopyridin-4-yl)morpholine (**13**)

A mixture of KSCN (48 mmol, 4.7 g) and compound **12** (37 mmol, 9 g) in acetic acid was stirred at 80 °C for 2h. Solvent was removed under reduced pressure, and the residue was purified with flash column chromatography on silica gel to afford compound **8** (5.1 g, 52% yield). Yellow solid, R_f_: 0.4 (EtOAc/Petroleum ether = 1:1), m.p.: 177–179 °C. ^1^H NMR (400 MHz, DMSO-*d6*) δ 8.29 (d, *J* = 6.1 Hz, 1H), 7.32 (d, *J* = 6.2 Hz, 1H), 3.72–3.64 (m, 4H), 3.35–3.29 (m, 4H). ^13^C NMR (101 MHz, DMSO-*d6*) δ 151.5, 150.2, 149.8, 130.5, 113.4, 109.4, 65.5, 50.2. HRMS (ESI): *m/z* (M + H^+^) calcd for C_10_H_11_N_4_O_3_S, 267.0546, found: 267.0541. IR (ATR): 3109, 2985, 2861, 1585, 1504, 1312, 1209, 1108, 961, 822 cm^−1^.

#### 3.2.3. Synthesis of 7-morpholinothiazolo[5,4-*b*]pyridin-2-amine (**14**)

A mixture of Fe powder (75.2 mmol, 4.2 g) and compound **13** (18.8 mmol, 5 g) in acetic acid was stirred at 60 °C for 2h and filtered. The filtrate was concentrated under reduced pressure, and the residue was purified with flash column chromatography on silica gel to afford compound **9** (2.44 g, 55% yield). Colorless solid, R_f_: 0.4 (DCM/MeOH = 20:1). m.p.: 230–232 °C. ^1^H NMR (400 MHz, DMSO-*d6*) δ 7.89 (d, *J* = 5.5 Hz, 1H), 7.55 (s, 2H), 6.65 (d, *J* = 5.6 Hz, 1H), 3.78–3.70 (m, 4H), 3.56–3.50 (m, 4H). ^13^C NMR (101 MHz, DMSO-*d6*) δ 162.1, 155.6, 146.0, 143.0, 135.3, 107.0, 66.0, 48.3. HRMS (ESI): *m/z* (M + H^+^) calcd for C_10_H_13_N_4_OS, 237.0805, found: 237.0807. IR (ATR): 3346, 3121, 2961, 1659, 1558, 1297, 1113, 973, 799, 700 cm^−1^.

#### 3.2.4. Synthesis of 4-(2-bromothiazolo[5,4-*b*]pyridin-7-yl)morpholine (**15**)

A mixture of CuBr_2_ (15 mmol, 3.4 g), tert-butyl nitrite (20 mmol, 2.0 g) and compound **14** (10 mmol, 2.4 g) in acetonitrile (20 mL) was stirred at room temperature for 2h. Water (20 mL) was added, and aqueous layer was extracted by DCM (20 mL × 2). The organic layer was combined and washed with brine and dried over anhydrous MgSO_4_. DCM was then removed under reduced pressure and the residue was purified with flash column chromatography on silica gel to afford the compound **10** (1.2 g, 40% yield. Colorless solid, R_f_: 0.4 (EtOAc/Petroleum ether = 1:1). m.p.: 168–170 °C. ^1^H NMR (400 MHz, DMSO-*d6*) δ 8.23 (d, *J* = 5.8 Hz, 1H), 6.88 (d, *J* = 5.9 Hz, 1H), 3.82–3.74 (m, 8H). ^13^C NMR (101 MHz, DMSO-*d6*) δ 161.1, 148.8, 148.2, 134.2, 132.7, 106.9, 65.8, 48.3. HRMS (ESI): *m/z* (M + H^+^) calcd for C_10_H_11_BrN_3_OS, 299.9801, found: 299.9802. IR (ATR): 3014, 2948, 2865, 1568, 1463, 1259, 1120, 1008, 956, 803, 669 cm^−1^.

#### 3.2.5. General Procedure for the Synthesis of Aryl Borates **18a**–**18f**

Sulfonyl chloride (1.2 equiv.) was added to a mixture of compound **16** (20 mmol), DMAP (2 mmol) and pyridine (30 mmol) in the DCM (50 mL), and the reaction; mixture was stirred at room temperature overnight. Solvent was then removed under reduced pressure and the residue was purified with flash column chromatography on silica gel to give the compound **17a**–**17f**. KOAc (25 mmol) was added to a mixture of PdCl_2_(dppf) (0.63 mmol), compound **17a**–**17f** (12.5 mmol) and bis(pinacolato)diboron (15 mmol) in 1,4-dioxane (25 mL), and the reaction mixture was stirred at 100 °C for 5 h. Solvent was removed under reduced pressure, and DCM (20 mL) was added. Then the mixture was filtered, and the filtrate was concentrated under reduced pressure and the residue was purified with flash column chromatography on silica gel to give the compound **18a**–**18f**.

*2,4-Difluoro-N-(2-methoxy-5-(4,4,5,5-tetramethyl-1,3,2-dioxaborolan-2-yl)pyridin-3-yl)benzenesulfonamide (**18a**)*. Colorless solid (3.5 g, 66% yield), m.p.: 163–165 °C. ^1^H NMR (400 MHz, DMSO-*d6*) δ 10.18 (s, 1H), 8.21 (s, 1H), 7.72 (s, 1H), 7.71–7.65 (m, 1H), 7.60–7.52 (m, 1H), 7.22–7.16 (m, 1H), 3.62 (s, 3H), 1.29 (s, 12H). ^13^C NMR (101 MHz, DMSO-*d6*). δ 165.0 (dd, *J* = 255.2, 12.1 Hz), 160.2, 159.3 (dd, *J* = 258.6, 14.1 Hz), 150.9, 141.0, 131.8 (d, *J* = 11.1 Hz), 125.0 (dd, *J* = 15.2, 4.0 Hz), 119.2, 116.7, 111.8 (dd, *J* = 22.2, 4.0 Hz), 105.7 (t, *J* = 26.3 Hz), 84.0, 53.4, 24.6. HRMS (ESI): *m/z* (M + H^+^) calcd for C_18_H_22_BF_2_N_2_O_5_S, 427.1305, found: 427.1314. IR (ATR): 3264, 2989, 2883, 1600, 1406, 1340, 1139, 1019, 969, 850, 670 cm^−1^.

*2-Chloro-4-fluoro-N-(2-methoxy-5-(4,4,5,5-tetramethyl-1,3,2-dioxaborolan-2-yl)pyridin-3-yl)benzenesulfonamide (**18b**)*. Colorless solid (3.4 g, 61% yield), m.p.: 138–140 °C. ^1^H NMR (400 MHz, DMSO-*d6*) δ 10.07 (s, 1H), 8.18 (d, *J* = 1.6 Hz, 1H), 7.85 (dd, *J* = 8.8, 6.0 Hz, 1H), 7.73 (dd, *J* = 8.7, 2.5 Hz, 1H), 7.68 (d, *J* = 1.5 Hz, 1H), 7.34 (td, *J* = 8.5, 2.5 Hz, 1H), 3.63 (s, 3H), 1.28 (s, 12H). ^13^C NMR (101 MHz, DMSO-*d6*) δ 163.8 (d, *J* = 255.9 Hz), 159.9, 150.6, 140.1, 134.5 (d, *J* = 3.4 Hz), 133.2 (d, *J* = 11.6 Hz), 132.9 (d, *J* = 10.3 Hz), 119.5, 119.2 (d, *J* = 24.7 Hz), 117.1, 114.5 (d, *J* = 21.9 Hz), 84.0, 53.4, 24.6. HRMS (ESI): *m/z* (M + H^+^) calcd for C_18_H_22_BClFN_2_O_5_S, 443.1010, found: 443.1001. IR (ATR): 3288, 3103, 2981, 1586, 1341, 1154, 908, 850, 679 cm^−1^.

*5-Chloro-N-(2-methoxy-5-(4,4,5,5-tetramethyl-1,3,2-dioxaborolan-2-yl)pyridin-3-yl)thiophene-2-sulfonamide (**18c**)*. Colorless solid (3.6 g, 67% yield), m.p.: 156–158 °C. ^1^H NMR (400 MHz, DMSO-*d6*) δ 10.21 (s, 1H), 8.24 (s, 1H), 7.73 (s, 1H), 7.29 (d, *J* = 4.1 Hz, 1H), 7.20 (d, *J* = 4.1 Hz, 1H), 3.71 (s, 3H), 1.29 (s, 12H). ^13^C NMR (101 MHz, DMSO-*d6*) δ 160.4, 151.2, 140.4, 139.4, 135.5, 132.5, 128.2, 119.9, 117.2, 84.5, 54.1, 25.1. HRMS (ESI): *m/z* (M + H^+^) calcd for C_16_H_21_BClN_2_O_5_S_2_, 431.0668, found: 431.0680. IR (ATR): 3251, 3088, 2924, 1599, 1397, 1344, 1253, 1153, 992, 852, 679 cm^−1^.

*N-(2-Methoxy-5-(4,4,5,5-tetramethyl-1,3,2-dioxaborolan-2-yl)pyridin-3-yl)methanesulfonamide (**18d**)*. Colorless solid (2.4 g, 58% yield), ^1^H NMR (400 MHz, DMSO-*d6*) δ 9.23 (s, 1H), 8.20 (s, 1H), 7.77 (s, 1H), 3.93 (d, *J* = 3.6 Hz, 3H), 3.01 (s, 3H), 1.29 (s, 12H). ^13^C NMR (101 MHz, DMSO-*d6*) δ 158.8, 149.24, 137.0, 121.0, 116.6, 83.9, 73.5, 53.8, 40.4, 24.9, 24.6. HRMS (ESI): *m/z* (M + H^+^) calcd for C_13_H_22_BN_2_O_5_S, 329.1337, found: 329.1318. IR (ATR): 3263, 2979, 1603, 1497, 1392, 1256, 1138, 970, 850, 771, 670 cm^−1^.

*2,4-Difluoro-N-(2-methoxy-5-(4,4,5,5-tetramethyl-1,3,2-dioxaborolan-2-yl)phenyl)benzenesulfonamide (**18e**)*. Colorless solid (3.3 g, 62% yield), m.p.: 150–152 °C. ^1^H NMR (400 MHz, DMSO-*d6*) δ 9.83 (s, 1H), 7.64 (dd, *J* = 14.9, 8.4 Hz, 1H), 7.57–7.47 (m, 3H), 7.16 (td, *J* = 8.6, 2.1 Hz, 1H), 6.92 (d, *J* = 8.2 Hz, 1H), 3.49 (s, 3H), 1.29 (s, 12H). ^13^C NMR (101 MHz, DMSO-*d6*) δ 164.8 (dd, *J* = 254.5, 12.1 Hz), 159.4 (dd, *J* = 258.6, 13.1 Hz), 156.5, 134.8, 134.5, 131.8 (d, *J* = 11.1 Hz), 125.3 (dd, *J* = 14.1, 4.0 Hz), 123.7, 120.1, 111.4 (dd, *J* = 22.2, 3.0 Hz), 111.3, 105.5 (t, *J* = 30.3 Hz), 83.6, 55.2, 24.7. HRMS (ESI): *m/z* (M + H^+^) calcd for C_19_H_23_BF_2_NO_5_S, 426.1353, found: 426.1369. IR (ATR): 3300, 2925, 2850, 1601, 1340, 1258 1167, 1129, 850, 669 cm^−1^.

#### 3.2.6. General Procedure for the Synthesis of Target Compounds **19a**–**19f**

K_2_CO_3_ aqueous solution (2N, 0.39 mL) was added to a mixture of PdCl_2_(dppf) (0.03 mmol), compound **15** (0.26 mmol) and Aryl borate **18** (0.31 mmol) in 1,4-dioxane (2.5 mL), and the reaction mixture was stirred at 100 °C for 5h. Solvent was removed under reduced pressure, and DCM (20 mL) was added. Then the mixture was filtered, and the filtrate was concentrated under reduced pressure and the residue was purified with flash column chromatography on silica gel to give compound **19**. ^1^H NMR and ^13^CNMR spectra of the compounds **19a**–**19f**, and the HPLC spectra of compound **19a** can be found in the Supplementary Materials.

*2,4-Difluoro-N-(2-methoxy-5-(7-morpholinothiazolo[5,4-b]pyridin-2-yl)pyridin-3-yl)benzenesulfonamide (**19a**)*. Colorless solid (103 mg, 76% yield), R_f_: 0.5 (DCM/MeOH = 30:1), m.p.: 175–177 °C. ^1^H NMR (400 MHz, DMSO-*d6*) δ 10.52 (s, 1H), 8.62 (d, *J* = 2.1 Hz, 1H), 8.21 (d, *J* = 5.7 Hz, 1H), 8.11 (d, *J* = 2.1 Hz, 1H), 7.80 (dd, *J* = 14.9, 8.5 Hz, 1H), 7.64–7.56 (m, 1H), 7.25 (td, *J* = 8.6, 2.1 Hz, 1H), 6.86 (d, *J* = 5.7 Hz, 1H), 3.88–3.78 (m, 8H), 3.75 (s, 3H). ^13^C NMR (101 MHz, DMSO-*d6*) δ 165.1 (dd, *J* = 255.5 Hz, 12.1 Hz), 159.4, 159.3 (dd, *J* = 258.6 Hz, 14.1 Hz), 158.9, 157.2, 149.3, 147.9, 142.7, 135.2, 131.8 (d, *J* = 11.1 Hz), 131.2, 124.8 (dd, *J* = 14.1 Hz, 3.0 Hz), 123.4, 120.5, 112.1 (dd, *J* = 22.2 Hz, 3.0 Hz), 106.4, 105.9 (t, *J* = 26.3 Hz), 65.9, 54.0, 48.5. HRMS (ESI): *m/z* (M + H^+^) calcd for C_22_H_20_F_2_N_5_O_4_S_2_, 520.0919, found: 520.0909. IR (ATR): 3183, 2922, 2854, 1602, 1423, 1261, 1149, 971, 864, 715, 668 cm^−1^.

*2-Chloro-4-fluoro-N-(2-methoxy-5-(7-morpholinothiazolo[5,4-b]pyridin-2-yl)pyridin-3-yl)benzenesulfonamide (**19b**)*. Colorless solid (102 mg, 73% yield), R_f_: 0.5 (DCM/MeOH = 30:1), m.p.: 188–190 °C. ^1^H NMR (400 MHz, DMSO-*d6*) δ 10.45 (s, 1H), 8.60 (d, *J* = 2.1 Hz, 1H), 8.20 (d, *J* = 5.7 Hz, 1H), 8.07 (d, *J* = 2.1 Hz, 1H), 7.97 (dd, *J* = 8.9, 5.9 Hz, 1H), 7.77 (dd, *J* = 8.7, 2.4 Hz, 1H), 7.40 (td, *J* = 8.5, 2.5 Hz, 1H), 6.86 (d, *J* = 5.7 Hz, 1H), 3.88–3.78 (m, 8H), 3.77 (s, 3H). ^13^C NMR (101 MHz, DMSO-*d6*) δ 163.9 (d, *J* = 255.5 Hz), 159.3, 158.6, 157.2, 149.3, 148.0, 142.4, 135.2, 134.3 (d, *J* = 3.0 Hz), 133.1 (d, *J* = 6.1 Hz), 133.0 (d, *J* = 4.0 Hz), 130.3, 123.3, 120.9, 119.4 (d, *J* = 26.3 Hz), 114.7 (d, *J* = 22.2 Hz), 106.4, 65.9, 54.0, 48.5. HRMS (ESI): *m/z* (M + H^+^) calcd for C_22_H_20_ClFN_5_O_4_S_2_, 536.0624, found: 536.0612. IR (ATR): 3376, 3008, 2921, 1568, 1471, 1341, 1253, 1157, 977, 803, 661 cm^−1^.

*5-Chloro-N-(2-methoxy-5-(7-morpholinothiazolo[5,4-b]pyridin-2-yl)pyridin-3-yl)thiophene-2-sulfonamide (**19c**)*. Colorless solid (95 mg, 70% yield), R_f_: 0.5 (DCM/MeOH = 30:1), m.p.: 116–118 °C. ^1^H NMR (400 MHz, DMSO-*d6*) δ 10.56 (s, 1H), 8.63 (d, *J* = 1.9 Hz, 1H), 8.21 (d, *J* = 5.6 Hz, 1H), 8.14 (d, *J* = 2.0 Hz, 1H), 7.44 (d, *J* = 4.1 Hz, 1H), 7.25 (d, *J* = 4.1 Hz, 1H), 6.87 (d, *J* = 5.7 Hz, 1H), 3.91–3.80 (m, 11H). ^13^C NMR (101 MHz, DMSO-*d6*) δ 159.3, 158.5, 157.4, 149.3, 148.0, 142.3, 138.8, 135.4, 135.3, 132.4, 129.4, 128.1, 123.4, 121.0, 106.5, 66.0, 54.2, 48.5. HRMS (ESI): *m/z* (M + H^+^) calcd for C_20_H_20_ClN_5_O_4_S_3_, 524.0282, found: 524.0285. IR (ATR): 3350 3015 2921, 1566, 1406, 1341, 1256, 1149, 976, 802, 676 cm^−1^.

*N-(2-Methoxy-5-(7-morpholinothiazolo[5,4-b]pyridin-2-yl)pyridin-3-yl)methanesulfonamide (**19d**)*. Colorless solid (73 mg, 67% yield), R_f_: 0.5 (DCM/MeOH = 30:1),m.p.: 230–232 °C. ^1^H NMR (400 MHz, DMSO-*d6*) δ 9.56 (s, 1H), 8.63 (d, *J* = 2.2 Hz, 1H), 8.24–8.20 (m, 2H), 6.89 (d, *J* = 5.7 Hz, 1H), 4.02 (s, 3H), 3.92–3.80 (m, 8H), 3.13 (s, 3H). ^13^C NMR (101 MHz, DMSO-*d6*) δ 159.4, 157.7, 157.6, 149.3, 148.0, 140.9, 135.3, 128.1, 123.5, 122.4, 106.5, 65.9, 54.3, 48.5, 40.6. HRMS (ESI): *m/z* (M + H^+^) calcd for C_17_H_20_N_5_O_4_S_2_, 422.0951, found: 422.0944. IR (ATR): 3215, 2998, 2882, 1585, 1464, 1393, 1287, 1137, 1003, 841, 771, 684 cm^−1^.

*2,4-Difluoro-N-(2-methoxy-5-(7-morpholinothiazolo[5,4-b]pyridin-2-yl)phenyl)benzenesulfonamide (**19e**)*. Colorless solid (101 mg, 75% yield), R_f_: 0.5 (DCM/MeOH = 30:1), m.p.: 195–197 °C. ^1^H NMR (400 MHz, DMSO-*d6*) δ 10.15 (s, 1H), 8.19 (d, *J* = 5.6 Hz, 1H), 7.84–7.80 (m, 2H), 7.74 (dd, *J* = 14.9, 8.5 Hz, 1H), 7.61–7.54 (m, 1H), 7.21 (t, *J* = 7.5 Hz, 1H), 7.11 (d, *J* = 8.4 Hz, 1H), 6.86 (d, *J* = 5.7 Hz, 1H), 3.89–3.79 (m, 8H), 3.62 (s, 3H). ^13^C NMR (101 MHz, DMSO-*d6*) δ 165.0 (dd, *J* = 253.5 Hz, 14.1 Hz), 160.0, 159.3, 159.2 (dd, *J* = 258.6 Hz, 15.2 Hz), 155.6, 149.3, 147.7, 135.6, 131.8 (d, *J* = 1.1 Hz), 126.6, 125.5, 125.3, 125.1 (dd, *J* = 14.1 Hz, 4.0 Hz), 124.7, 112.5, 111.8 (dd, *J* = 23.2 Hz, 4.0 Hz), 106.5,105.8 (t, *J* = 26.3 Hz), 65.9, 55.8, 48.5. HRMS (ESI): *m/z* (M + H^+^) calcd for C_23_H_21_F_2_N_4_O_4_S_2_, 519.0967, found: 519.0970. IR (ATR): 3268, 2922, 2844, 1567, 1426, 1336, 1272, 1122, 969, 816, 670 cm^−1^.

*2,4-Difluoro-N-(5-(7-morpholinothiazolo[5,4-b]pyridin-2-yl)pyridin-3-yl)benzenesulfonamide (**19f**)*. Colorless solid (57 mg, 45% yield), R_f_: 0.3 (DCM/MeOH = 30:1). m.p.: 99–101 °C. ^1^H NMR (400 MHz, DMSO-*d6*) δ 11.35 (s, 1H), 8.87 (s, 1H), 8.47 (d, *J* = 2.0 Hz, 1H), 8.23 (d, *J* = 5.7 Hz, 1H), 8.08 (s, 1H), 8.00 (dd, *J* = 14.8, 8.5 Hz, 1H), 7.61–7.54 (m, 1H), 7.32 (t, *J* = 8.4 Hz, 1H), 6.89 (d, *J* = 5.7 Hz, 1H), 3.92–3.80 (m, 8H). ^13^C NMR (101 MHz, DMSO-*d6*) δ 165.5 (dd, *J* = 256.5, 12.1 Hz), 159.6, 159.0 (dd, *J* = 258.6, 14.1 Hz), 156.9, 149.6, 148.5, 142.9, 142.5, 135.3, 134.6, 132.4 (d, *J* = 10.1 Hz), 129.1, 123.4 (dd, *J* = 14.1, 3.0 Hz), 123.2, 112.7 (dd, *J* = 22.2, 4.0 Hz), 106. (t, *J* = 25.3 Hz), 65.9, 48.5. HRMS (ESI): *m/z* (M + H^+^) calcd for C_21_H_18_F_2_N_5_O_3_S_2_, 490.0814, found: 490.0805. IR (ATR): 3000, 2928, 1557, 1476, 1425, 1343, 1266, 1068, 961, 846, 669 cm^−1^.

## 4. Conclusions

In summary, we have designed and synthesized a series of 2-pyridyl, 4-morpholinyl substituted thiazolo[5,4-*b*]pyridines by pharmacophore splicing of methoxyl pyridine and morpholinyl heterocyclic inhibitors. These molecules were evaluated for the inhibitory activity of kinases. It was found that compound **19a** exhibited nanomolar inhibitory activity against the three isoforms of PI3Kα, PI3Kγ, and PI3Kδ. Further docking study demonstrated that compound **19a** fit well into the ATP binding pocket of the PI3Kα kinase. Overall, these novel thiazolo[5,4-*b*]pyridines may be potentially used as the potent PI3K inhibitors for the treatment of disease mediated by the PI3K signaling pathway after further activity evaluation in vivo and optimization of their pharmacological properties.

## Figures and Tables

**Table 1:** SAR studies of 2-substitutions on the thiazolo[5,4-*b*]pyridine.

Compd	X	R^1^	R^2^	cLogP *^a^*	PSA *^a^*	PI3Kα (nm) *^b^*
**19a**	N	OMe	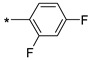	4.1	105.0	3.6
**19b**	N	OMe	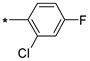	4.4	105.0	4.6
**19c**	N	OMe	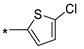	4.3	105.0	8.0
**19d**	N	OMe	Me	2.1	105.0	53
**19e**	CH	OMe	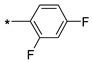	4.4	92.6	501
**19f**	N	H	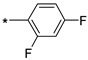	3.3	95.7	4.0

*^a^* Calculated from ChemBioDraw Ultra 14.0. *^b^* Mean of at least three separate experiments.

**Table 2:** Enzymatic inhibition by compound **19a** *^a^*.

Enzymatic Assay	IC_50_ (nM)
PI3Kɑ	3.6
PI3Kβ	34
PI3Kγ	1.6
PI3Kδ	2.9

*^a^* Mean of at least three separate experiments.

## Supplementary Materials

^1^H NMR and ^13^CNMR spectra of the compounds **19a**–**19f**, and the HPLC spectra of compound **19a**.
