# Supplementary material for: Identification of Novel Thiazolo[5,4-b]Pyridine Derivatives as Potent Phosphoinositide 3-Kinase Inhibitors
Source: Molecules. 2020 Oct 12;25(20):4630. doi: 10.3390/molecules25204630 (PMC7594053; doi:10.3390/molecules25204630)
Supplement: Supplementary file 1 [file molecules-25-04630-s001.pdf]

Supporting Information

Identification of Novel Thiazolo[5,4-b]Pyridine Derivatives as Potent Phosphoinositide 3-Kinase Inhibitors

Liang Xia [1,2], Yan Zhang[1,2], Jingbo Zhang[1,2], Songwen Lin [1,2], Kehui Zhang [1,2], Hua Tian[1,2], Yi Dong [1,2,]* and Heng Xu [1,2,]*

[1] State Key Laboratory of Bioactive Substance and Function of Natural Medicines, Institute of Materia Medica, Chinese Academy of Medical Sciences and Peking Union Medical College, Beijing 100050, China.

[2] Beijing Key Laboratory of Active Substances Discovery and Druggability Evaluation, Institute of Materia Medica, Chinese Academy of Medical Sciences and Peking Union Medical College, Beijing 100050, China

E-mail: dongyi@imm.ac.cn; xuheng@imm.ac.cn

Table of cotent

*^{1}H NMR, ^{13}C NMR, HPLC spectrum of 2,4-difluoro-N-(2-methoxy-5-(7-morpholinothiazolo[5,4-b] pyridin-2-yl)pyridin-3-yl)benzenesulfonamide **(19a)**……………………………………………………………S1-S3*

*^{1}H NMR, ^{13}C NMR, HPLC spectrum of 2-chloro-4-fluoro-N-(2-methoxy-5-(7-morpholinothiazolo [5,4-b]pyridin-2-yl)pyridin-3-yl)benzenesulfonamide (**19b**)…………………………………………………..S4- S6*

*^{1}H NMR, ^{13}C NMR, HPLC spectrum of (5-chloro-N-(2-methoxy-5-(7-morpholinothiazolo[5,4-b] pyridin-2-yl)pyridin-3-yl)thiophene-2-sulfonamide (**19c**)……………………………………………………..S7-S9*

*^{1}H NMR, ^{13}C NMR, HPLC spectrum of 4-(2-(6-methoxypyridin-3-yl)thiazolo[5,4-b]pyridin-7-yl) Morpholine (**19d**)……….S10-S12*

*^{1}H NMR, ^{13}C NMR, HPLC spectrum of 2,4-difluoro-N-(2-methoxy-5-(7-morpholinothiazolo[5,4-b] pyridin-2-yl)phenyl)benzenesulfonamide (**19e**)…………………………………………………………………S13-S15*

*^{1}H NMR, ^{13}C NMR, HPLC spectrum of 2,4-difluoro-N-(5-(7-morpholinothiazolo[5,4-b]pyridin-2-yl) pyridin-3-yl)benzenesulfonamide (**19f**)……………………………………………………………………………. S16-S18*

^{1}H NMR spectrum of **19a**

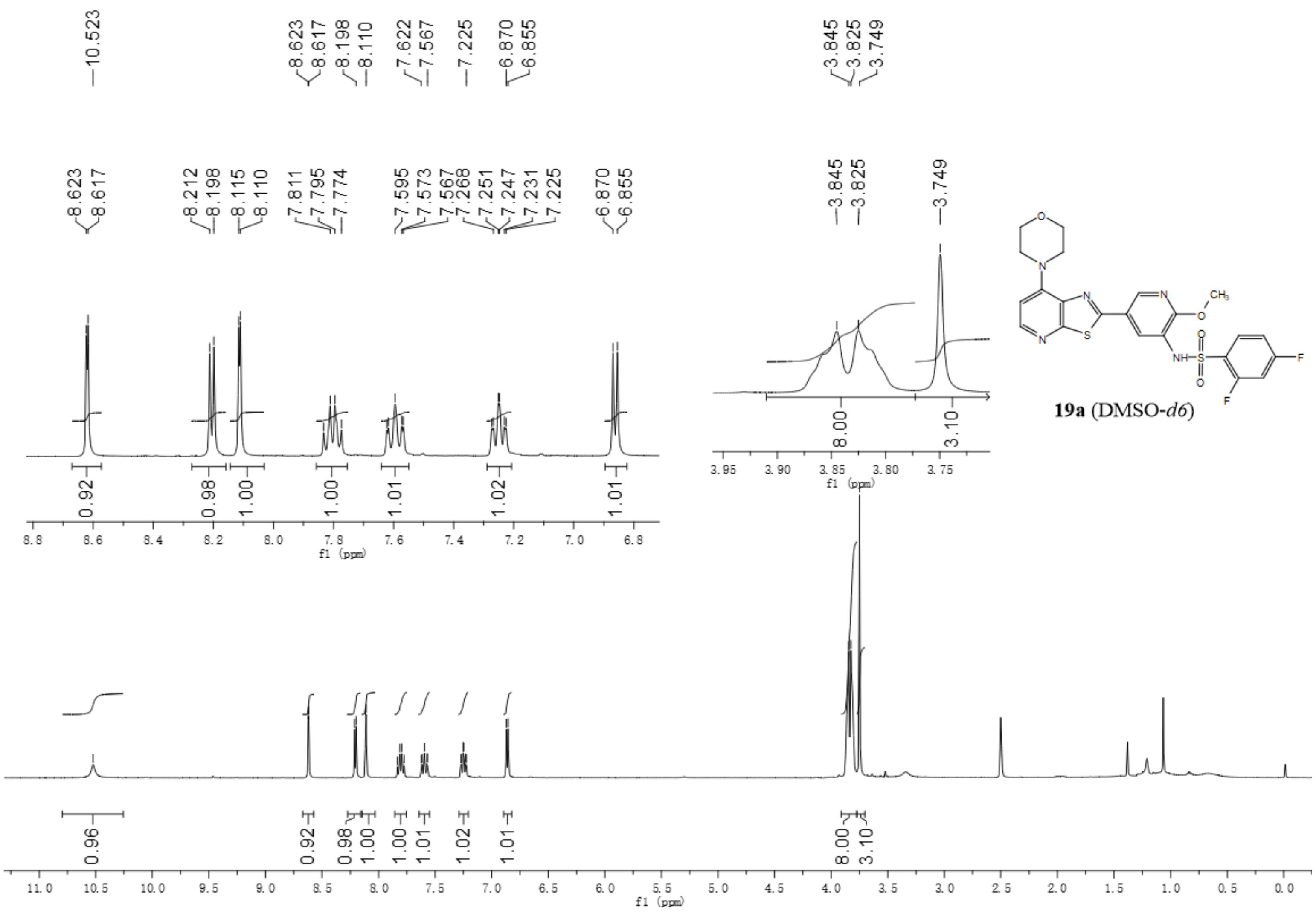

19a (DMSO-*d6*)

^{13}C NMR spectrum of **19a**

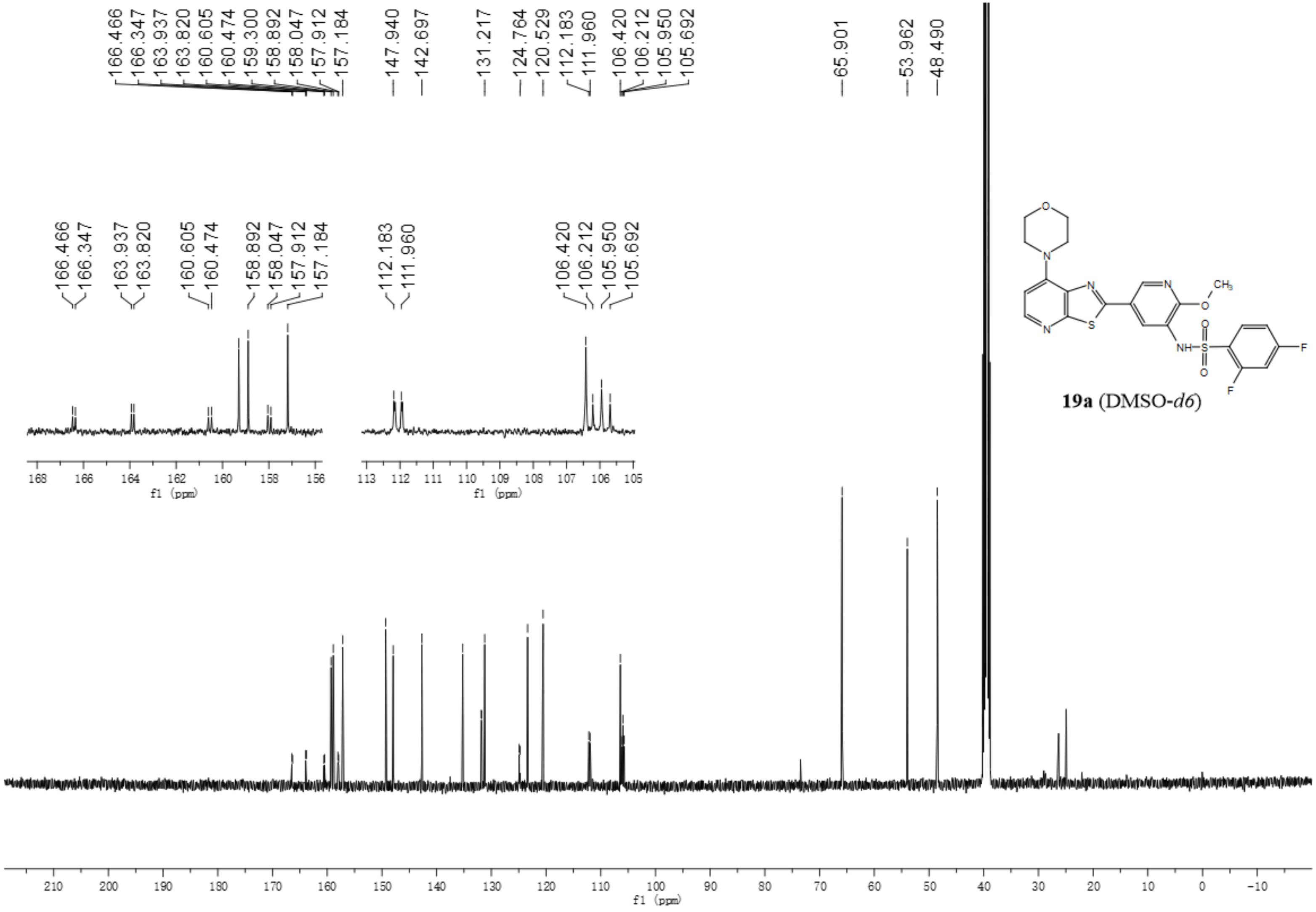

HPLC spectrum of **19a**

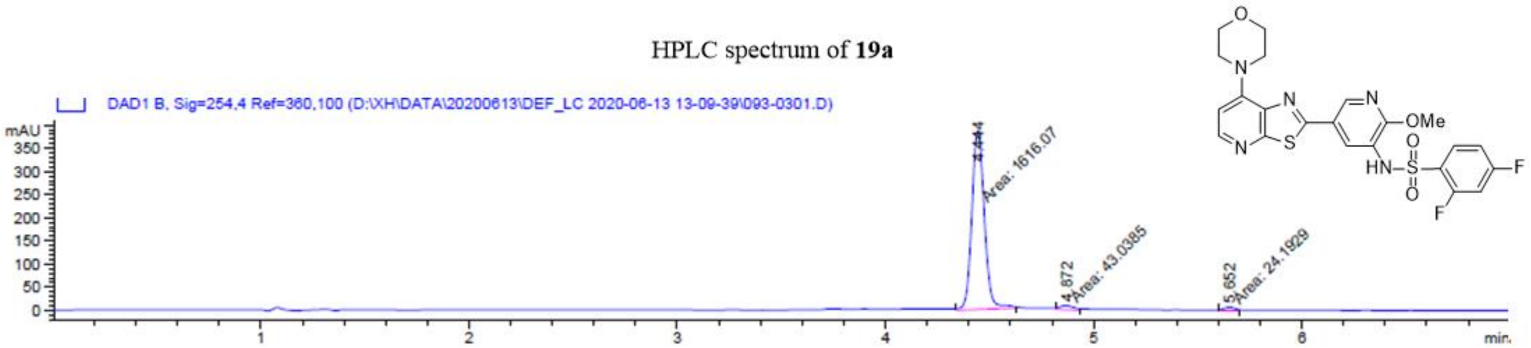

Signal 2: DAD1 B, Sig=254,4 Ref=360,100

Peak #	RetTime [min]	Type	Width [min]	Area [mAU*s]	Height [mAU]	Area %
1	4.444	MM	0.0692	1616.07092	388.99686	96.0060
2	4.872	MM	0.0801	43.03851	8.95479	2.5568
3	5.652	MM	0.0630	24.19288	6.39731	1.4372

^{1}H NMR spectrum of **19b**

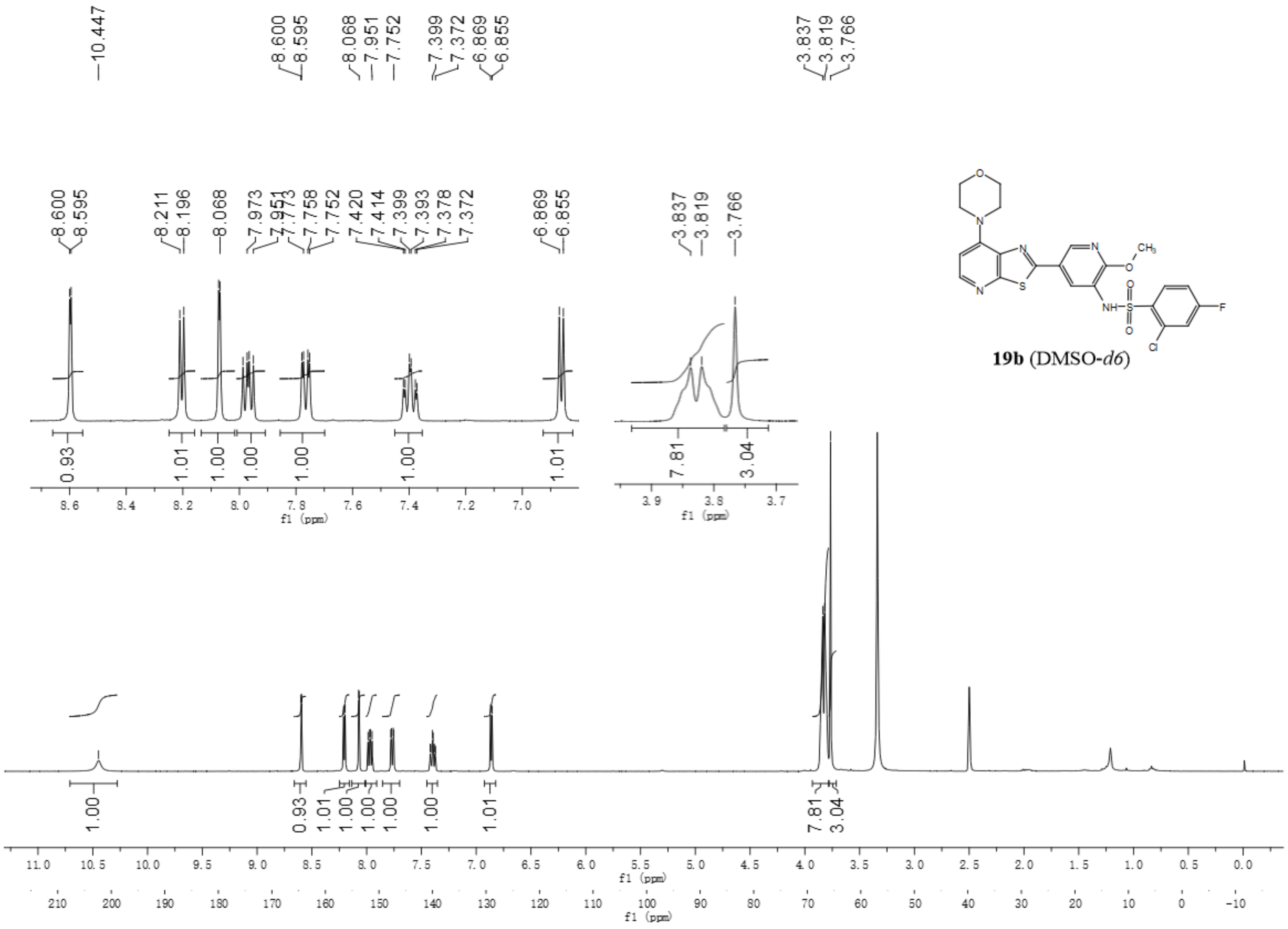

19b (DMSO-*d6*)

^{13}C NMR spectrum of **19b**

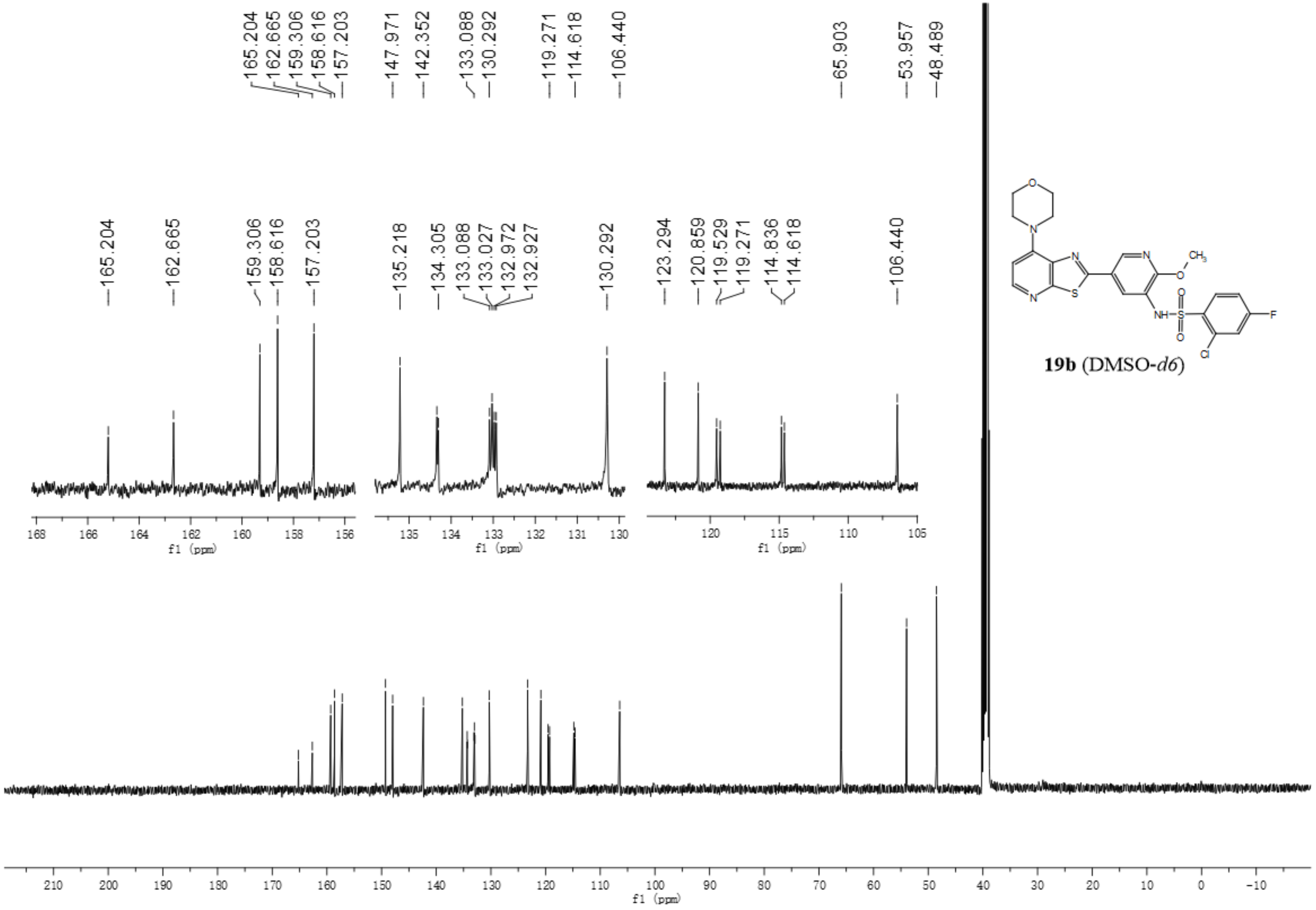

19b (DMSO-*d6*)

HPLC spectrum of **19b**

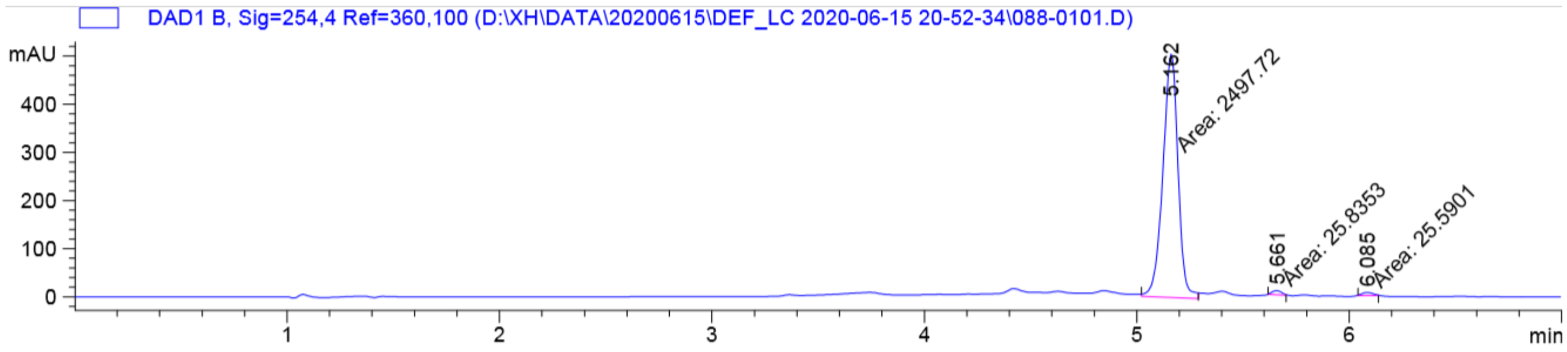

Signal 2: DAD1 B, Sig=254,4 Ref=360,100

Peak #	RetTime [min]	Type	Width [min]	Area [mAU*s]	Height [mAU]	Area %
1	5.162	MM	0.0821	2497.72070	506.81339	97.9826
2	5.661	MM	0.0516	25.83528	8.34479	1.0135
3	6.085	MM	0.0622	25.59011	6.85190	1.0039

^{1}H NMR spectrum of **19c**

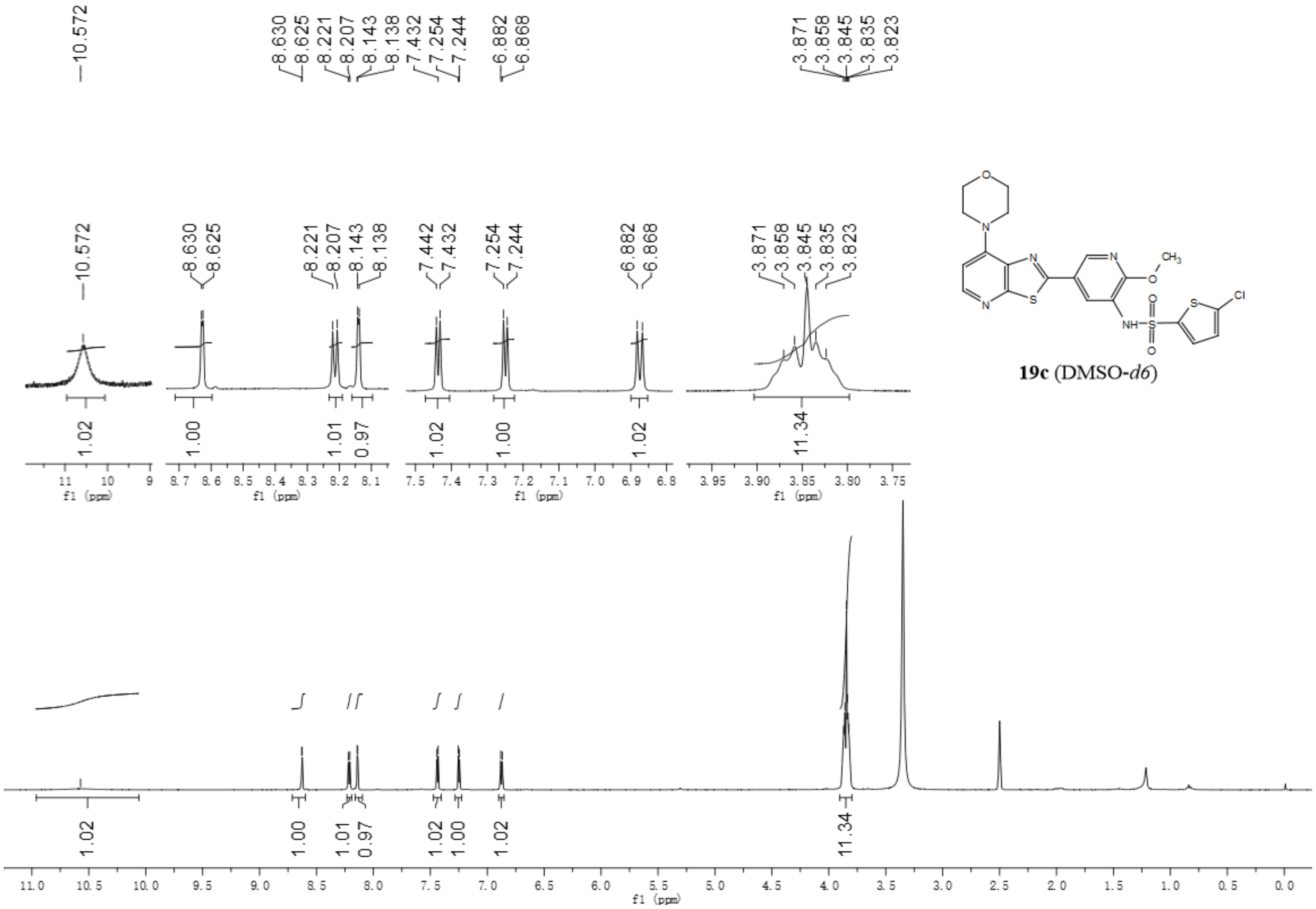

19c (DMSO-*d6*)

^{13}C NMR spectrum of **19c**

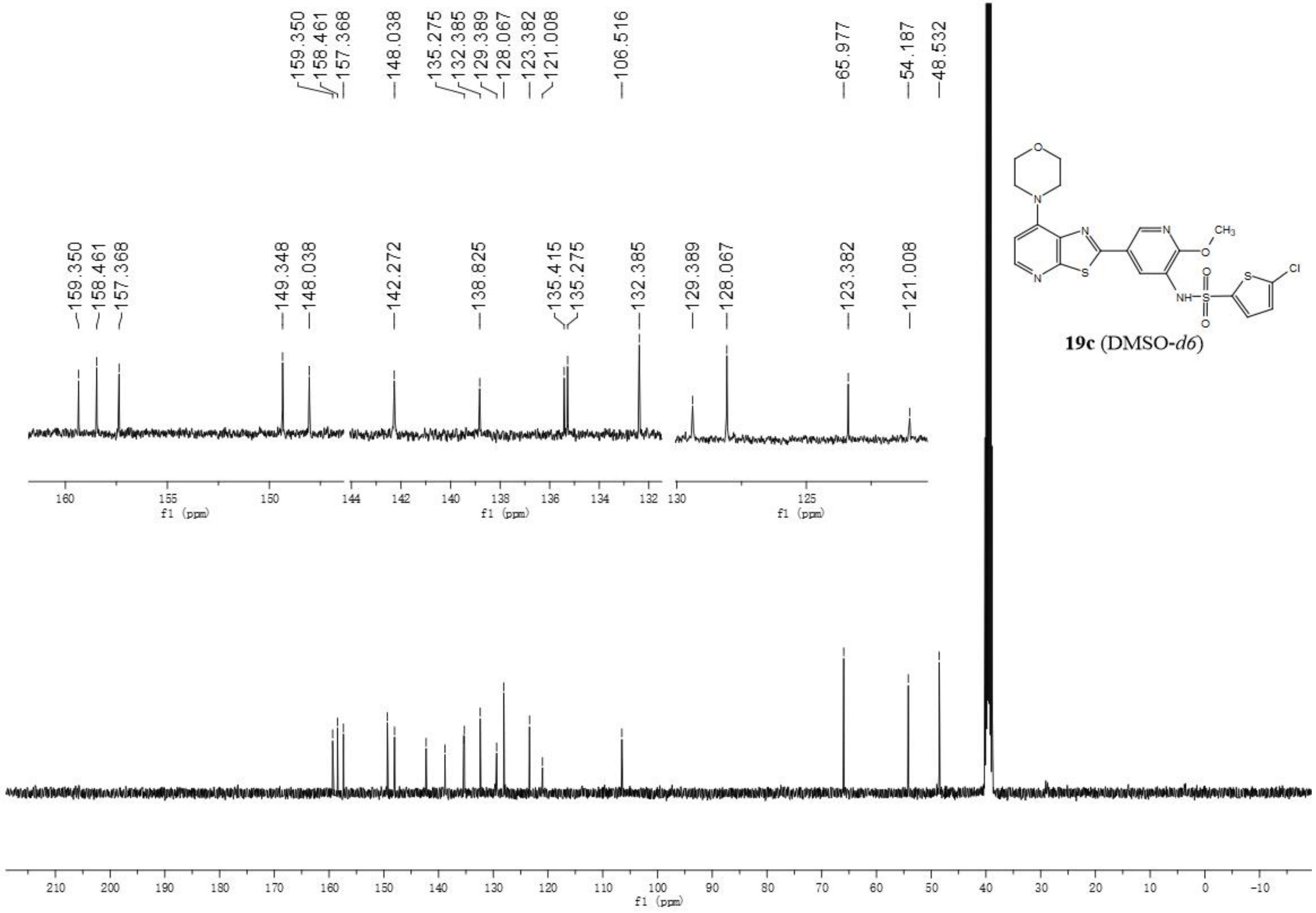

19c (DMSO-*d6*)

HPLC spectrum of **19c**

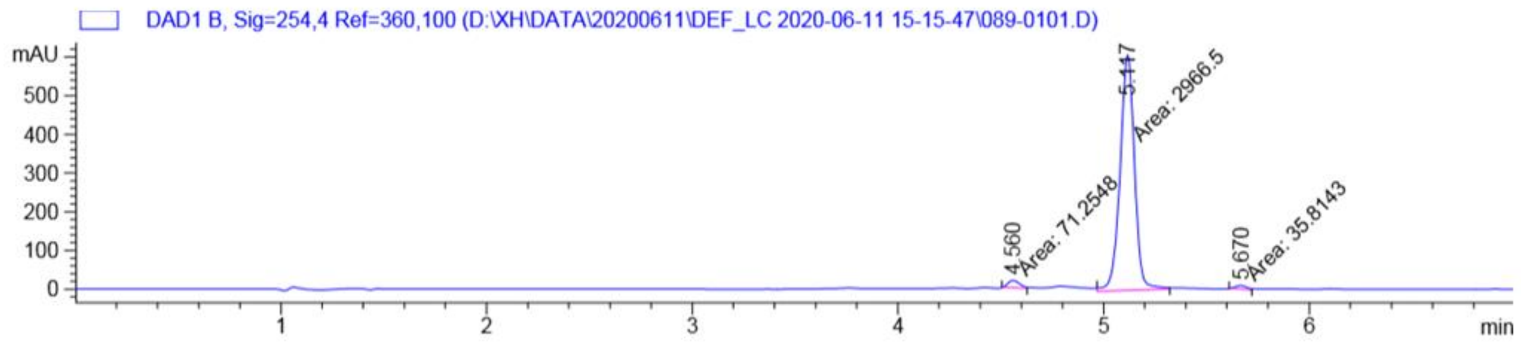

Signal 2: DAD1 B, Sig=254,4 Ref=360,100

Peak #	RetTime [min]	Type	Width [min]	Area [mAU*s]	Height [mAU]	Area %
1	4.560	MM	0.0651	71.25484	18.23404	2.3183
2	5.117	MM	0.0810	2966.49585	610.17664	96.5165
3	5.670	MM	0.0655	35.81426	9.11627	1.1652

[1] H NMR spectrum of **19d**

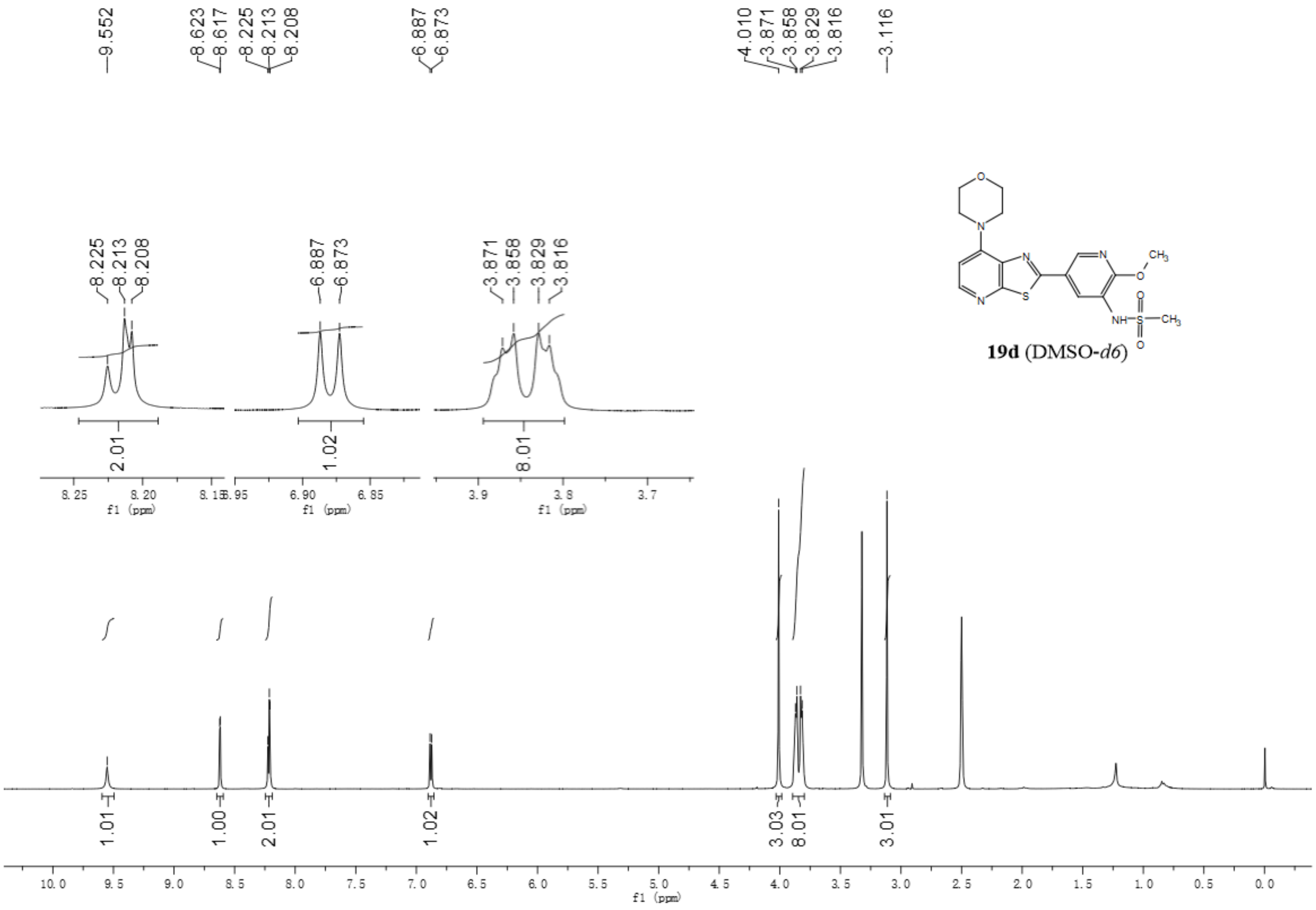

19d (DMSO-*d6*)

^{13}C NMR spectrum of **19d**

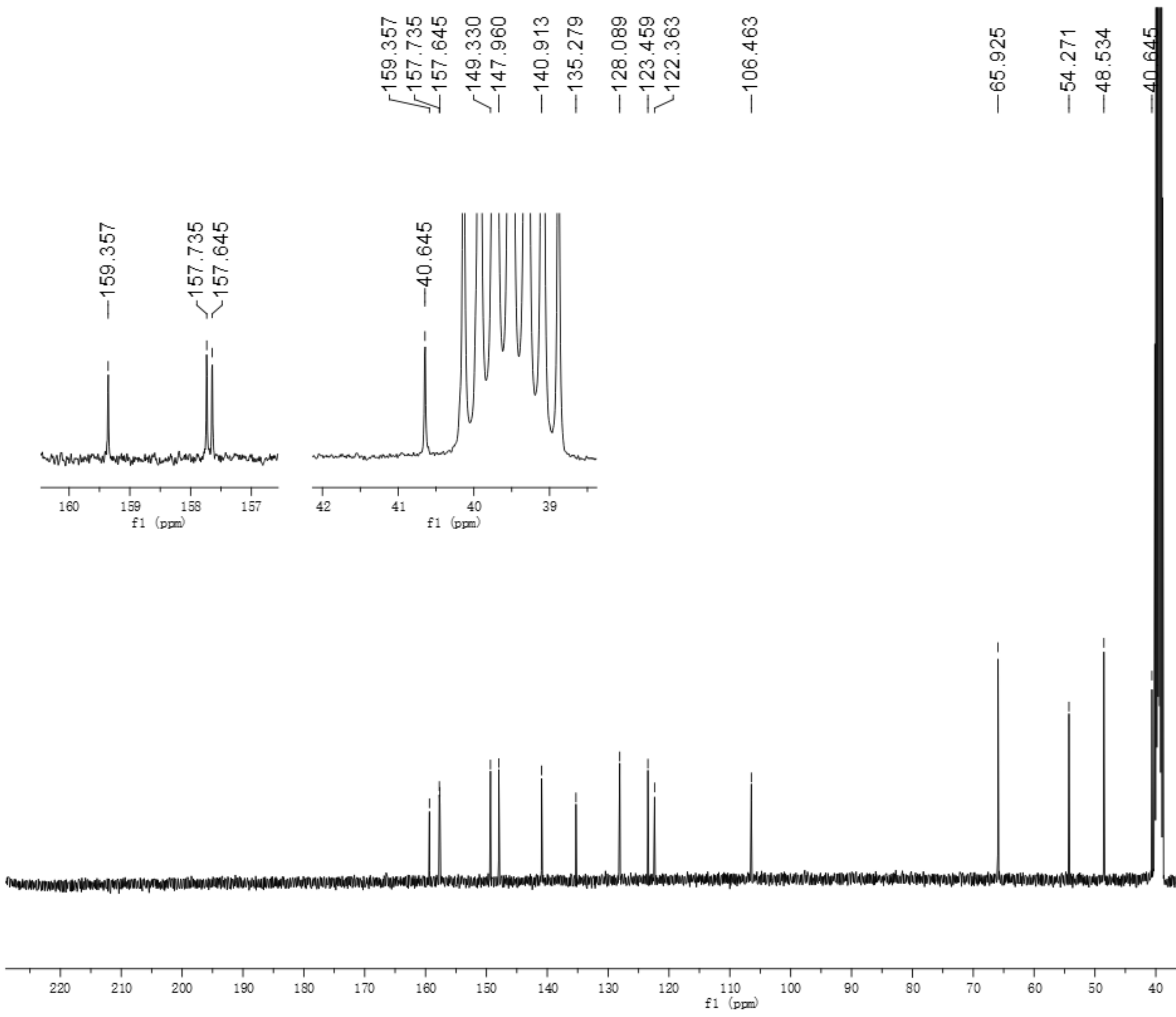

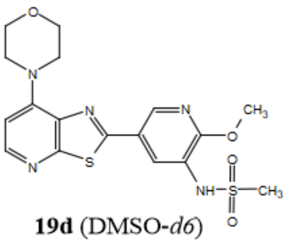

19d (DMSO-*d6*)

HPLC spectrum of **19d**

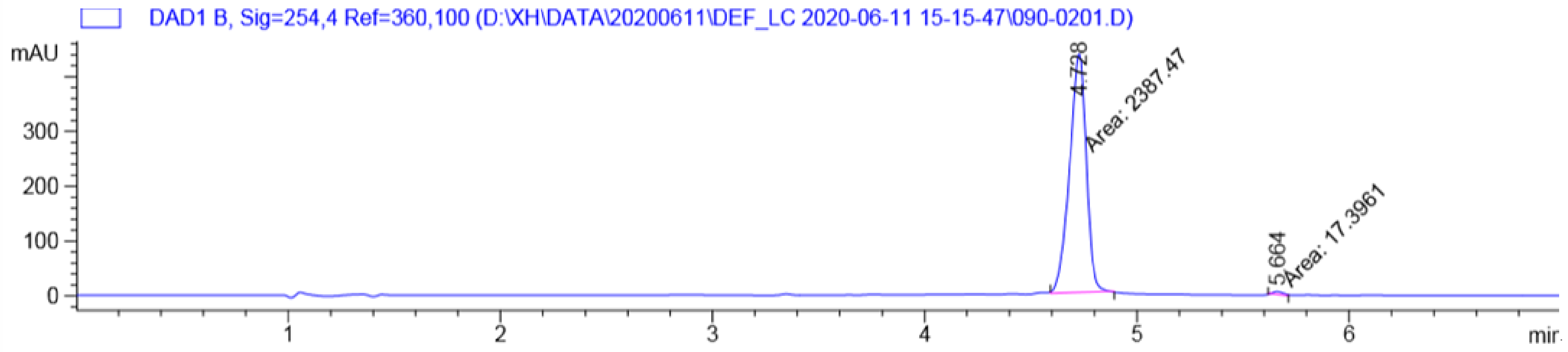

Signal 2: DAD1 B, Sig=254,4 Ref=360,100

Peak #	RetTime [min]	Type	Width [min]	Area [mAU*s]	Height [mAU]	Area %
1	4.728	MM	0.0910	2387.46973	437.42035	99.2766
2	5.664	MM	0.0545	17.39607	5.31610	0.7234

^1H NMR spectrum of **19e**

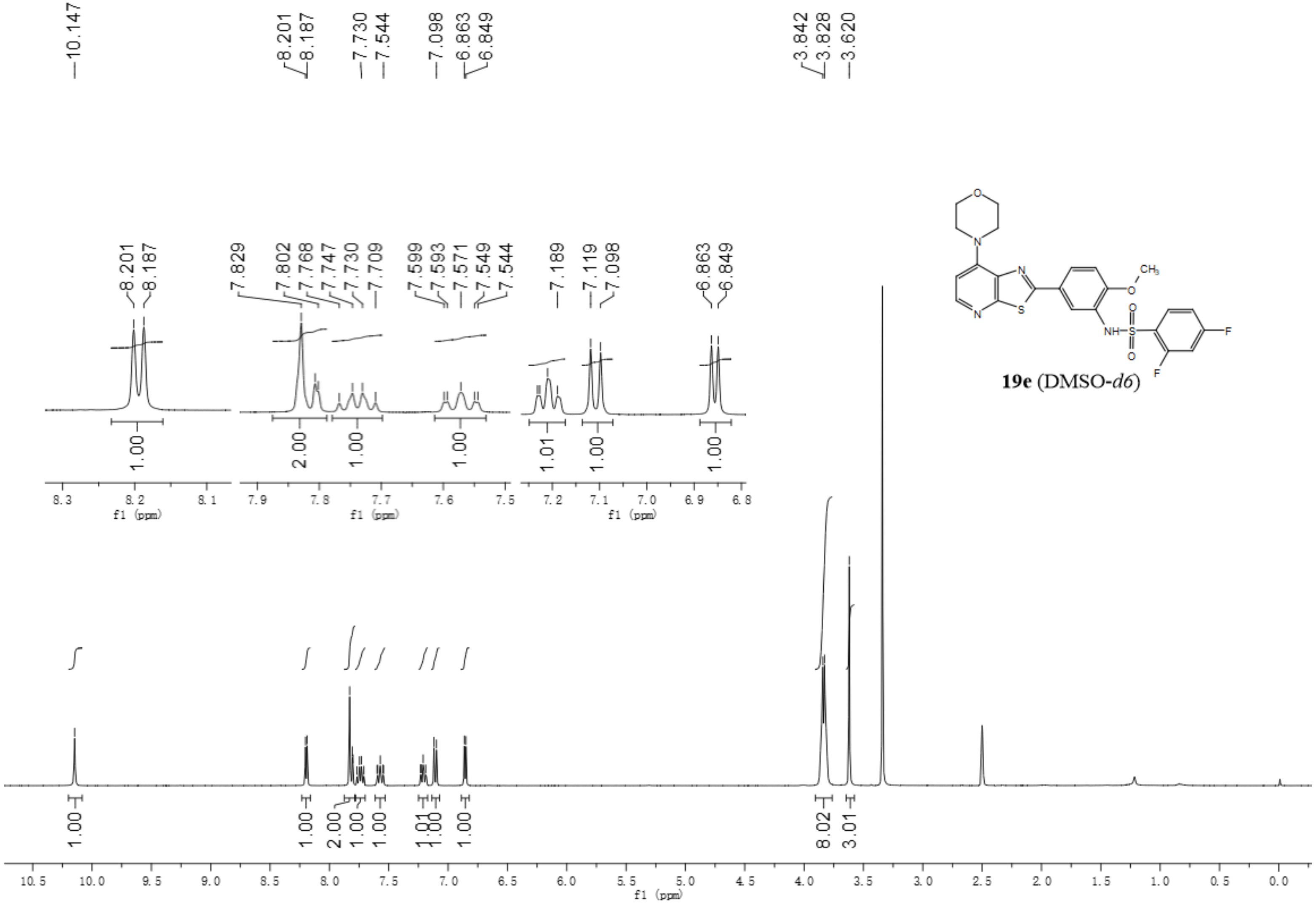

19e (DMSO-*d6*)

13 CNMR spectrum of **19e**

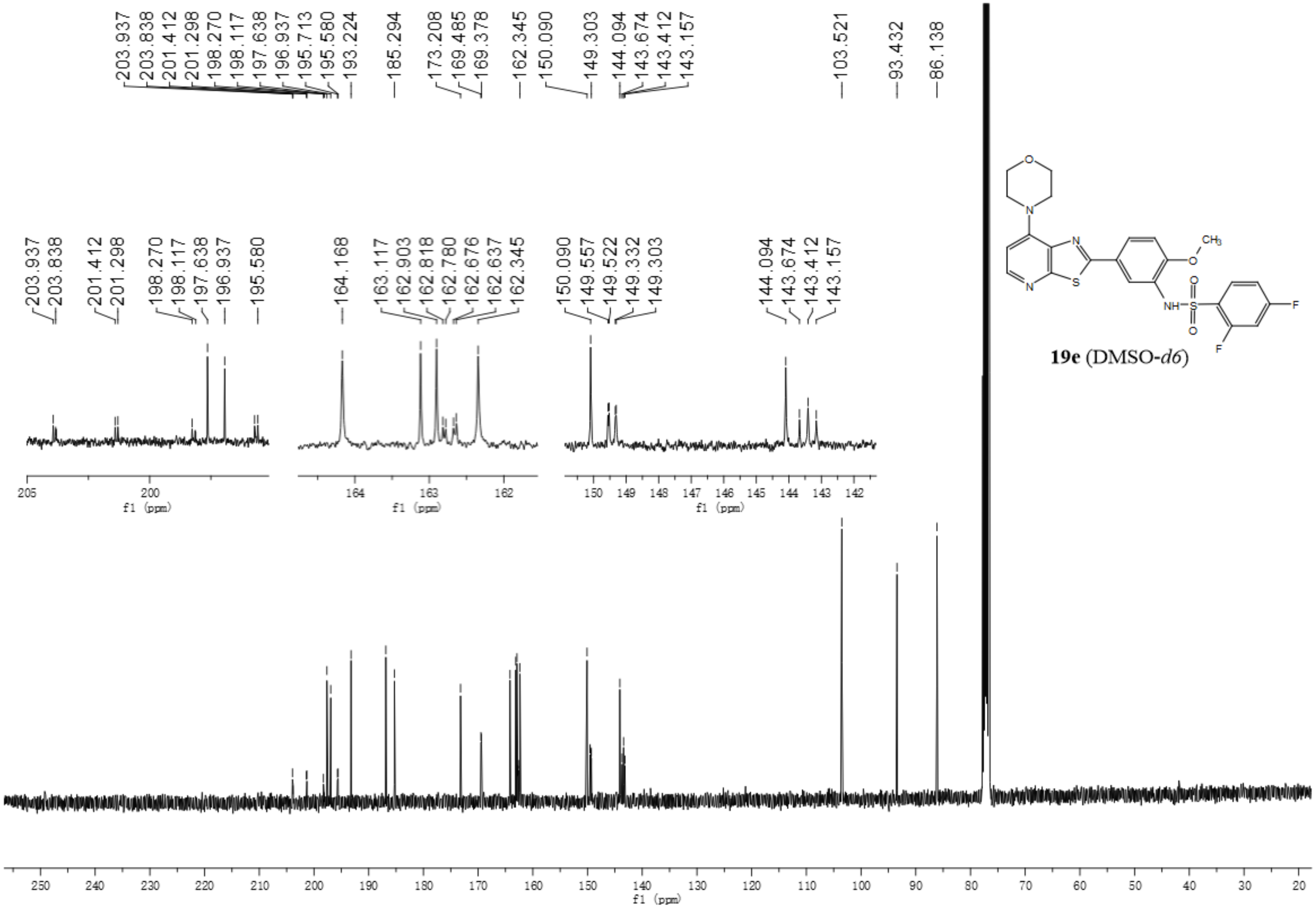

HPLC spectrum of **19e**

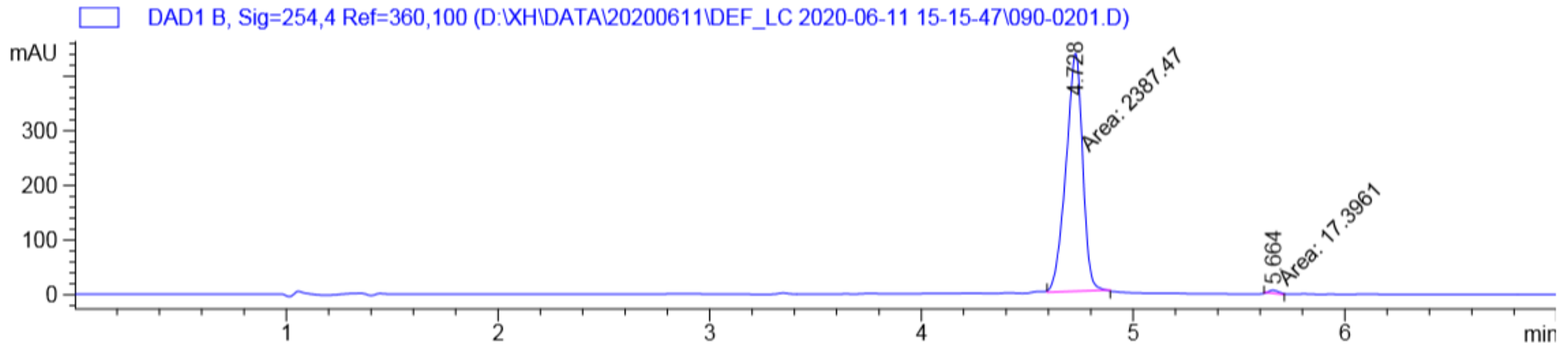

Signal 2: DAD1 B, Sig=254,4 Ref=360,100

Peak #	RetTime [min]	Type	Width [min]	Area [mAU*s]	Height [mAU]	Area %
1	4.728	MM	0.0910	2387.46973	437.42035	99.2766
2	5.664	MM	0.0545	17.39607	5.31610	0.7234

^{1}H NMR spectrum of **19f**

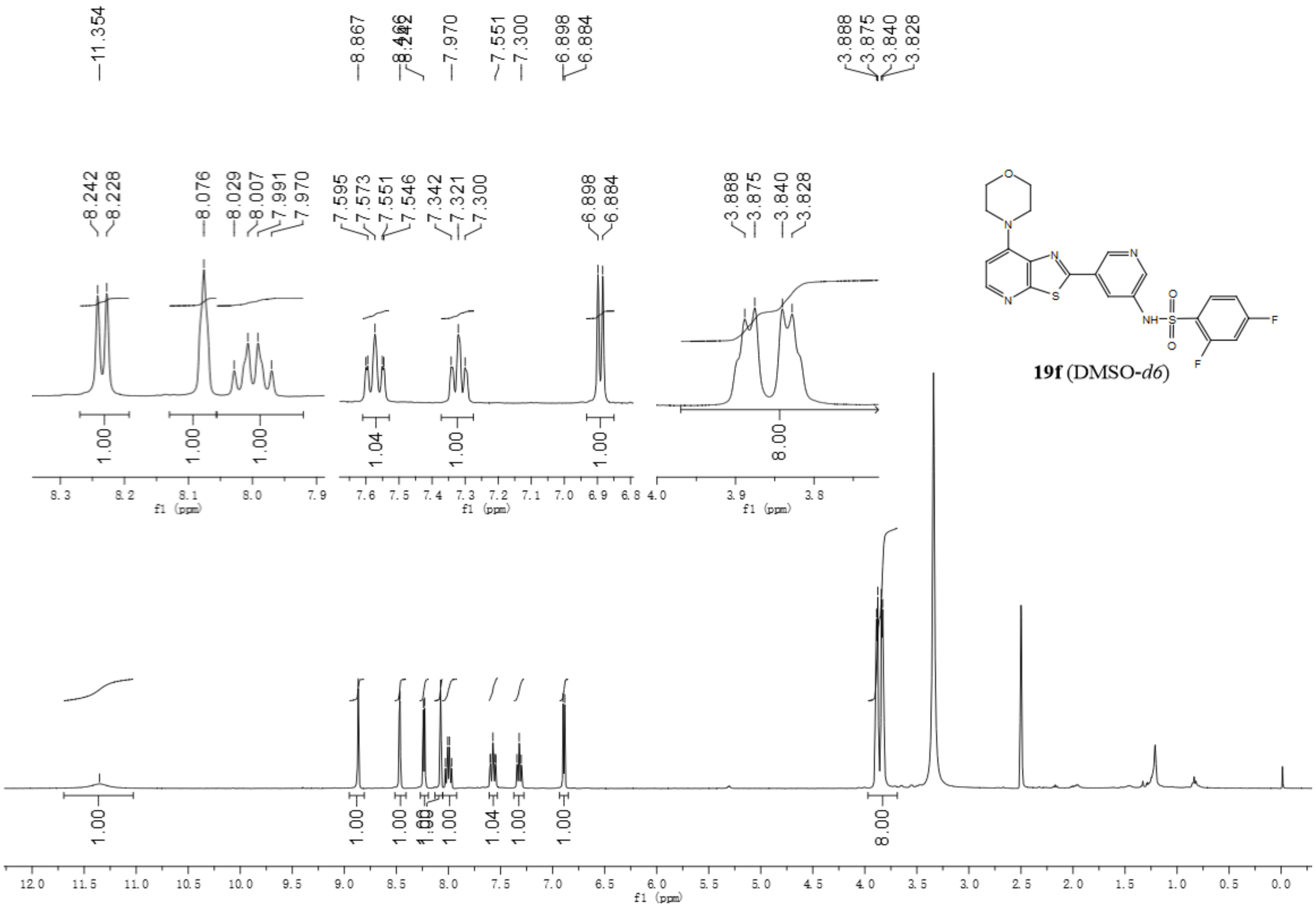

19f (DMSO-*d6*)

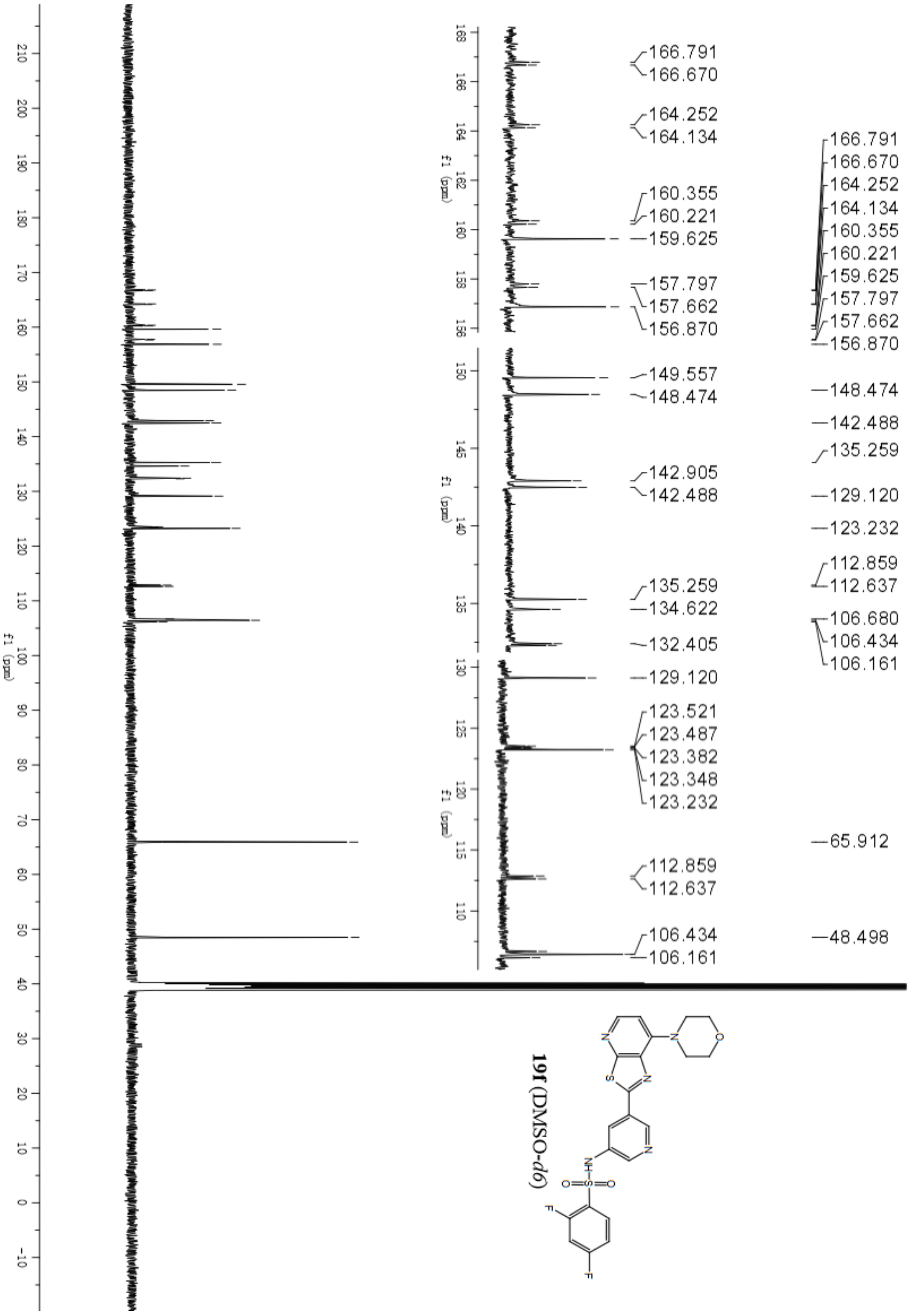

^{13}C NMR spectrum of **19f**

HPLC spectrum of **19f**

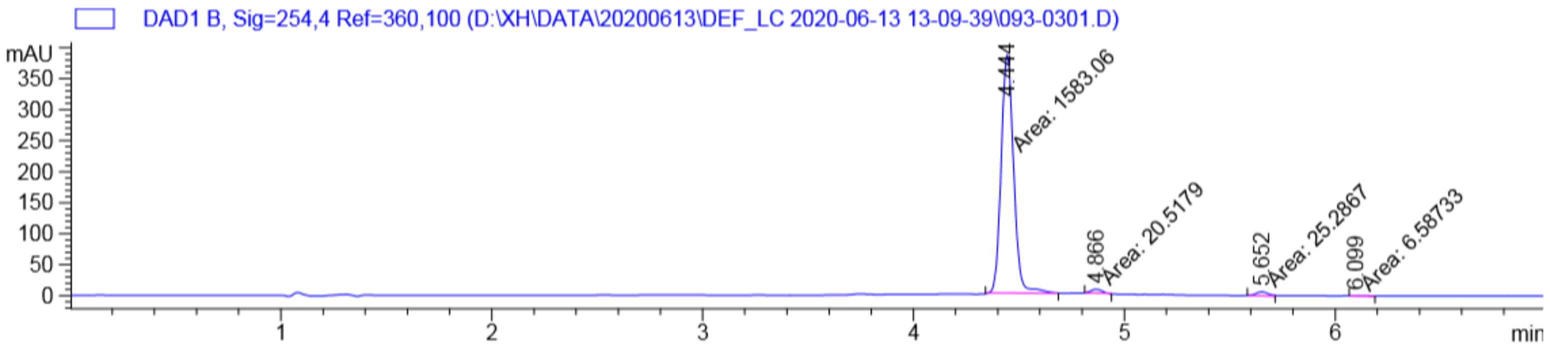

Signal 2: DAD1 B, Sig=254,4 Ref=360,100

Peak #	RetTime [min]	Type	Width [min]	Area [mAU*s]	Height [mAU]	Area %
1	4.444	MM	0.0684	1583.05725	385.95856	96.7965
2	4.866	MM	0.0591	20.51789	5.78970	1.2546
3	5.652	MM	0.0668	25.28674	6.31196	1.5462
4	6.099	MM	0.1068	6.58733	1.02797	0.4028
